# Functionalizing Graphene Oxide with Alkylamine by Gamma-ray Irradiation Method

**DOI:** 10.3390/nano7060135

**Published:** 2017-06-03

**Authors:** Noraniza Ahmad Daud, Buong Woei Chieng, Nor Azowa Ibrahim, Zainal Abidin Talib, Ernee Noryana Muhamad, Zurina Zainal Abidin

**Affiliations:** 1 Department of Chemistry, Faculty of Science, Universiti Putra Malaysia, 43400 UPM Serdang, Selangor, Malaysia; noranizaad@gmail.com (N.A.D.); norazowa@upm.edu.my (N.A.I.); ernee@upm.edu.my (E.N.M.); 2 Materials Processing and Technology Laboratory, Institute of Advanced Technology, Universiti Putra Malaysia, 43400 UPM Serdang, Selangor, Malaysia; 3 Department of Physics, Faculty of Science, Universiti Putra Malaysia, 43400 UPM Serdang, Selangor, Malaysia; zainalat@upm.edu.my; 4 Department of Chemical and Environmental Engineering, Faculty of Engineering, Universiti Putra Malaysia, 43400 UPM Serdang, Selangor, Malaysia; zurina@upm.edu.my

**Keywords:** graphene oxide, functionalization, alkylamine

## Abstract

A gamma-ray irradiation technique was used to functionalize graphene oxide (GO) with alkylamines of various alkyl chain lengths. Functionalization of the alkyl chains onto the GO was confirmed by nuclear magnetic resonance (^1^H NMR), Fourier transform infrared (FTIR), and X-ray diffraction (XRD). FTIR of the functionalized GO showed the appearance of significant peaks around 2960–2850 cm^−1^ (–CH_2_) which came from long alkyl chains, together with a peak around 1560–1450 cm^−1^, indicating the formation of C–NH–C. XRD showed an additional diffraction peak at a lower 2θ angle, indicating that the intercalation of the alkylamine was successful. The effects on the morphological and thermal properties of GO functionalized with alkyl chains of various lengths were investigated. Scanning electron microscopy (SEM) analysis showed an increase in surface roughness when the alkyl chain length was increased. The addition of alkyl chains on GO surfaces significantly improved the thermal stability of the GO, suggesting that these surfaces have great potential for use as a hydrophobic material in industry.

## 1. Introduction

Graphene has drawn a great deal of attention from scientific communities in recent years due to its excellent mechanical, thermal, optical and electronic properties. It’s a new class of two-dimensional carbon nanostructures with a single-layer sp^2^ network of atoms in a honeycomb lattice. It can be produced by the mechanical exfoliation of graphite, epitaxial growth, chemical vapour deposition (CVD), liquid-phase exfoliation [1], solvent–thermal synthesis [2], and the reduction of graphene oxide (GO). Among them, the reduction of GO is the most economic method for the synthesis of graphene and is based on the exfoliation of graphite into one-atom-thick sheets by oxidation, creating GO and the subsequent reduction of GO into a graphene sheet by reducing agents such as hydrazine, hydroquinone, vitamin C, sodium borohydride, dimethylformamide, urea and so on. The reduced GO approach almost achieves the highly desired properties of graphene such as variable electrical conductivity, but not completely.

On the other hand, GO is a graphene sheet with oxygen functional groups on its edges and basal plane, as proposed by Lerf and co-workers [3]. It is believed that carboxylic and carbonyl groups are at its edges, whereas phenol, hydroxyl, and epoxide groups are on its basal plane [4]. GO is an interesting derivative of a carbon material which has sparked a tremendous amount of attention from the science community in recent years due to its unique and amazing properties. It has been proposed for use in electronic devices, sensors, energy materials, hydrogen storage materials, and also as a value-added filler in composite materials. 

Brodie, in 1859, first demonstrated the synthesis of GO by adding a portion of potassium chlorate to a slurry of graphite in fuming nitric acid. In 1898, Staudenmaier improved on this protocol by using concentrated sulfuric acid as well as fuming nitric acid, and by adding the chlorate in multiple aliquots over the course of the reaction. In 1958, Hummers reported the method most commonly used today: oxidizing graphite by treatment with KMnO_4_ and NaNO_3_ in concentrated H_2_SO_4_. However, all three of these procedures involve the generation of the toxic or explosive gases NO_2_, N_2_O_4_ and ClO_2_. The various synthesis methods are summarized in Table 1. Nevertheless, the synthesis of graphene by these methods is fraught with problems, including defective graphene layers and difficulties in scale-up production. An alternative that mitigates the problems of graphene is the use of GO.

Nowadays, researchers are always interested in finding a filler material which promises to deliver strong and multifunctional properties even at low filler content. In fact, the excellent performance of GO as a filler does not only depend on its inherent properties, but more importantly on the compatibility of GO with the polymer matrices. So, the challenge to achieve a good dispersion and compatibilization becomes a major obstacle to these goals. The presence of moieties with oxygen functionalities on GO such as carboxyl, epoxide, and hydroxyl leads to a lower compatibility with non-polar polymer matrices. The introduction of hydrophobic groups on GO surfaces is an attractive objective, especially when aiming for compatibilization with non-polar material.

In 2006, Ruof et al. modified GO with isocyanate to reduce the hydrophilic properties of GO so that it could then be exfoliated in a polar aprotic solvent [5]. Afterwards, many researchers modified GO with octadecylamine (ODA) through various types of reactions. Mai et al. prepared functionalized GO with ODA via a nucleophilic substitution reaction to improve the dispersion of these materials with polystyrene matrices [6]. This method also increases the conductivity of the material to a point where it can be used as a good electrical conductor. Feng et al., prepared functionalized GO with long alkyl chains by simply using an amidation reaction and the result showed homogenous dispersion and an intimate adhesion to a polypropylene matrix [7]. Compton et al. also modified GO with hexylamine, followed by reduction processes to improve the conductivity of GO. This can be an attractive strategy for producing electrically conductive material [8]. Furthermore, Mei et al. modified GO with various alkylamines to switch the hydrophilic properties of GO to hydrophobic [9]. Based on a review of the literature, we see that the chemicals with long alkyl chains, such as ODA, the have been used for the modification of GO, not only change the hydrophilic properties of GO to hydrophobic, but also can improve other features such as the conductivity, dispersibility and solubility of GO.

Until now, there have been many different methods for the functionalization of GO, such as chemical methods that usually require a long reaction times, involve toxic reagents, and require with strict reaction conditions which limit large-scale production [8,10,11,12]. Therefore, a novel route that combines the economic benefits and convenience of chemical synthesis with a high reduction efficiency has been an import target for the preparation of functionalized GO. Recently, gamma-ray (γ-ray) irradiation has attracted attention among researchers not only because it is an environmentally friendly method, but also because it is feasible, cost effective, and can be performed at room temperature. However, the study of γ-ray-induced grafting on the surface of GO is still an emerging field. 

The irradiation of materials with ionizing radiation produces free radicals via excitation and ionization, which leads to the modification processes that cause the alteration of the physical, chemical, and biological properties of a product. Gamma-ray irradiation is known as a clean method for modifying the properties of carbon materials, promoting chemical reactions on their surfaces, and for modifying their functional groups [13,14]. Upon exposure to γ-ray irradiation, GO can be simply converted to reduced GO (rGO) via removal of the oxygen functionalities from its surfaces [15,16]. The thermal stability of the multi-walled carbon nanotubes (MWCNTs) can also be improved, simultaneously reducing the weight losses, and confirming the effectiveness of the method of chemical reduction by irradiation [17]. In addition, some researchers found that the γ-ray irradiation of carbon nanotube yarns improved the tensile strength and the modulus of the yarns due to the interactions between the individual nanotubes [18]. All these examples suggest that this method is probably the best alternative for the functionalization of materials, and can be extended to various combinations of conductive polymers to prepare new materials with desirable properties.

In this paper, we demonstrate that GO can be produced using an improved Hummers method without using NaNO_3_ and proceed with the functionalization of the GO with an alkylamine using a γ-ray irradiation technique. This method decreases the cost, time and environmental impact of GO production and functionalization. The effects of different alkyl chain lengths were investigated. 

## 2. Results and Discussion

Natural graphite was used as the starting material. The inter-graphene layers can be intercalated by various molecular species or ions, during which the interlayer spacing can change. We employed KMnO_4_ to oxidize the natural graphite powders. The yield (the weight of GO divided by weight of graphite powder) of GO produced was measured to be ~100%. 

### 2.1. X-ray Diffraction (XRD)

**Figure 1:**
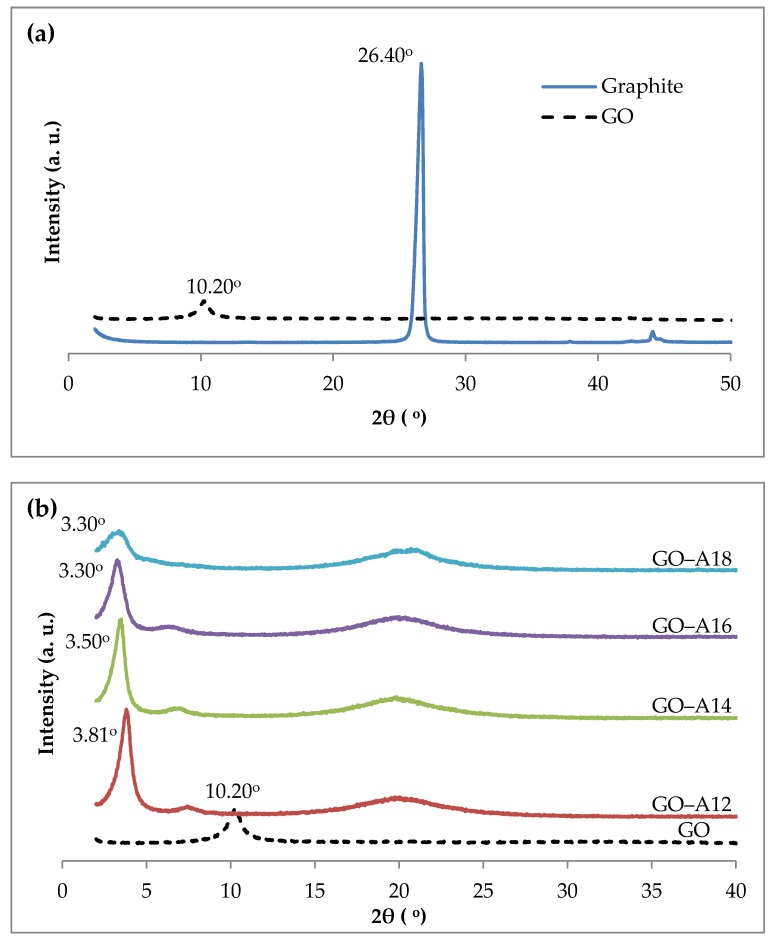
XRD spectra of (**a**) graphite and GO; and (**b**) functionalized GO.

The mechanism of GO production is mainly the generation of oxygen-containing groups on the graphene’s surface by an oxidation reaction. During the oxidation process, hydroxyl, carbonyl, epoxy or peroxy groups were bonded to the edges of the basal plane of the graphite. Simultaneously, carbon hydrolyzation occurred and the sp^2^ bonds changed to sp^3^ bonds. At the same time, H_2_O or SO_4_^2−^ ions could insert themselves into the graphene layer to increase the interlayer spacing. In Figure 1a of the XRD results, there is only one diffraction peak around 2θ = 26.40° in pristine graphite, a typical reflection peak (002) in graphite, and the d-spacing is about 0.334 nm. As oxidation proceeds, the intensity of this peak gradually weakened and finally disappeared. Simultaneously, the diffraction peak at about 10.20° appeared to correspond to an increase in the interlayer spacing from 0.334 nm of graphite to 0.824 nm of GO. The interlayer spacing of GO is increased due to the abundant presence of covalently-bound oxygen-containing functional groups on the graphene sheets resulting from the oxidation process. These abundant oxygen-containing functional groups weaken the Van der Waals interactions between the graphene sheets and thus increase the distance between the sheets [25,26,27,28]. The oxidation process itself also causes of the breaking of the graphite structure into smaller fragments [29]. Upon functionalization with the alkylamine, the interlayer spacing was further enlarged by the intercalation of the long-chain alkylamine between the graphene layers. XRD patterns of GO and various types of alkylamine-functionalized GO (GO–A8, GO–A12, GO–A18) are shown in Figure 1b. The interaction of the alkylamine with the GO may decrease the order of crystallinity of the graphite or deteriorate the degree of graphite crystallinity, which causes the main diffraction peak to become broader and shift to a slightly lower angle [30]. After functionalization, the peak was further shifted to 3.81° (GO–A12), 3.50° (GO–A14), and 3.30° (GO–A16 and GO–A18), corresponding to the expansion of the interlayer of the GO sheets from 0.824 nm to 2.325 nm (GO–A12), 2.525 nm (GO–A14) and 2.677 nm (GO–A16 and GO–A18), respectively. On the other hand, the broad peak around 18–24° arose due to the formation of reaggregated graphene sheets [12]. This change supports the successful functionalization of GO with alkylamine by the γ-ray irradiation technique.

### 2.2. Fourier Transform Infrared (FTIR)

**Figure 2:**
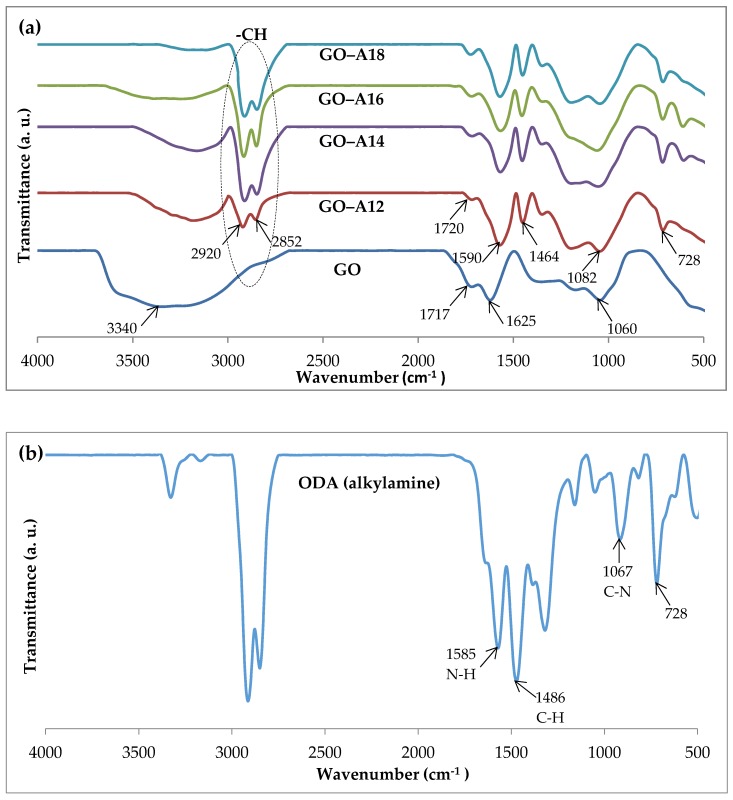
FTIR spectra of (**a**) GO and functionalized GO; and (**b**) octadecylamine (ODA).

FTIR analysis can be used as direct evidence for the functionalization of GO as it provides information about functional groups that are present in the sample. FTIR spectra of GO and functionalized GO are presented in Figure 2a. Generally, GO shows typical broad peaks at ~3340 cm^−1^ which is associated with hydroxyl groups, while the peak at 1717 cm^−1^ corresponds to carboxyl groups. The peak at 1625 cm^−1^ corresponds to a C=C bond in an aromatic ring and the peak at 1060 cm^−1^ is for C–O–C arranged in an epoxide ring [25,26,30]. This indicates that the solution of concentrated H_2_SO_4_ containing KMnO_4_ is capable of oxidizing graphite to GO with a high product yield, even without the assistance of NaNO_3_, as used in Hummers method. Thus, the FTIR data verifies the existence of oxygen-containing functional groups on GO. Upon functionalization with various alkylamines through γ-ray irradiation, the peaks around 2920–2852 cm^−1^ appeared, corresponding to the C–H stretching vibration of the alkyl chain (CH_2_), together with a peak around ~728 cm^−1^. Of note is that the absorbance of the –CH_2_ peaks increases as the length of the alkyl chain of increases, for example, GO–A18 compared to GO–A12. This result is comparable with the studied by Shanmugharaj et al. [12] and Yan et al. [31].

The peak at 1590 cm^−1^ of functionalized GO is probably due to the N–H bend, while the same peak appears at 1585 cm^−1^ in pristine ODA (shown in Figure 2b). The peak at 1082 cm^−1^ for functionalized GO is probably due to the shift of the C–N stretch at 1067 cm^−1^ in the parent amine, ODA. The peak at 1486 cm^−1^ of ODA belongs to the C–H bend, and it moves to 1464 cm^−1^ in functionalized GO. The spectral shift observed might have resulted from the functionalization of GO with the alkylamine. The bands at 1717 cm^−1^ and 1060 cm^−1^ corresponding to carboxyl and epoxide were weakened after functionalization and a new peak at 1082 cm^−1^ (C–N) appeared, indicating the formation of C–NH–C bonds [11]. Generally, the γ-ray irradiation method enables simultaneous functionalization and reduction of GO [14,15,30,32,33]. The decrease in the intensity of the bands at ~1717 (C=O), 1625 (C=C) and 1060 (C–O–C) cm^−1^ could be related to the creation of water or labile oxygen groups during γ-ray irradiation, as reported by Anson et al. [34].

### 2.3. Thermogravimetric Analysis (TGA)

**Figure 3:**
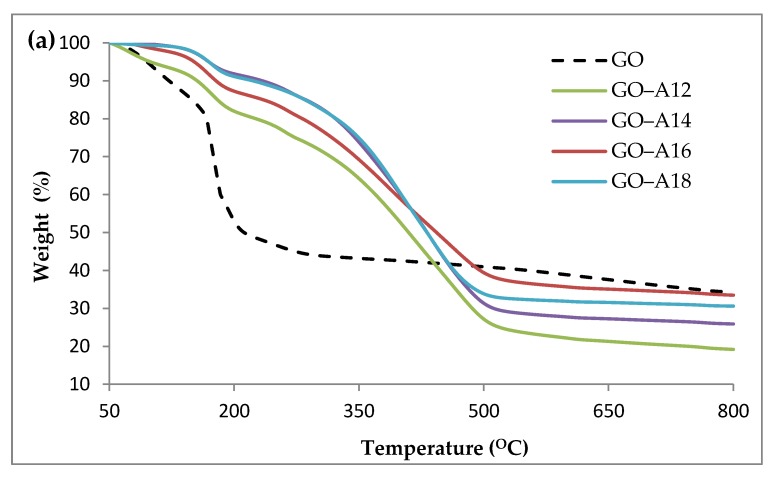

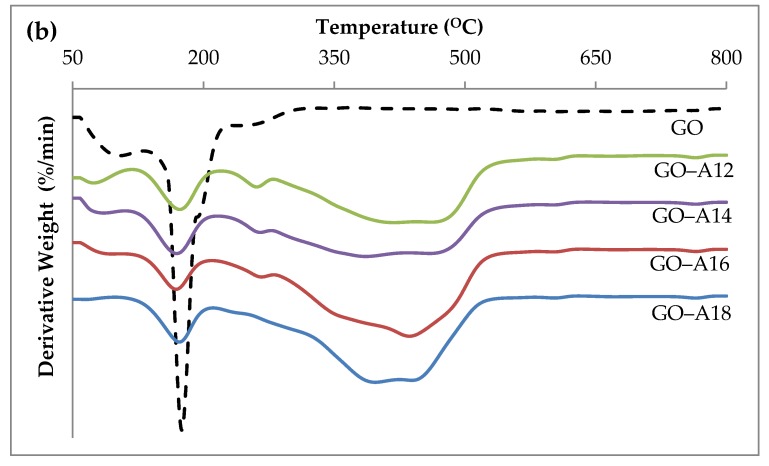
(**a**) TG; and (**b**) DTG thermograms of GO and functionalized GO.

TGA was used to investigate the thermal stability of the samples. Figure 3 depicts the (a) thermogravimetric (TG) and (b) derivative thermogravimetric (DTG) thermograms of GO and functionalized GO. Pristine GO showed a major weight loss of about 80% in the temperature range of 110–230 °C corresponding to the decomposition of labile oxygen-containing functionalities and illustrating a lower thermal stability. A low level of decomposition of the GO around 260 °C is due to more stable oxygen-containing functional groups and the bulk pyrolysis of the carbon skeleton [35]. The functionalized GO showed less than 10% weight loss below 100 °C, which indicated an enhanced hydrophobicity that minimized the amount of adsorbed water [12], compared to the pristine GO which usually exhibited a weight loss of about ~18%, corresponding to the evaporation of the trapped water molecules in the GO [36]. The major weight loss of the functionalized GO of about ~60% in the range of 300–550 °C was attributed to the thermal decomposition of the alkylamine chains. The DTG results suggested there were multiple processes occurring in the decomposition of the alkylamine in the region. The lower degradation temperature in the region may be attributed to the loss of small fragments of alkylamine that were physically adsorbed on the GO surfaces (free alkylamine). The physically adsorbed alkylamines are weaker than the alkylamines that had chemically grafted onto the GO surface. These, therefore, had a lower decomposition temperature [12]. The higher degradation temperature was supported by the degradation of the chemically-grafted alkylamine that intercalated between the GO sheets [11]. The thermal stability of functionalized GO increased substantially below 550 °C with increasing chain length of the grafted alkylamine. 

### 2.4. Nuclear Magnetic Resonance (NMR)

**Figure 4:**
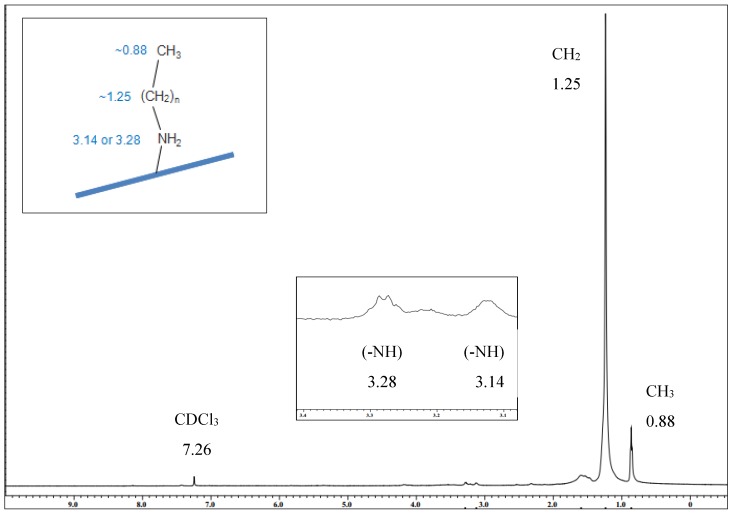
^1^H NMR spectrum of GO–A18 in CDCl_3_.

NMR was used to elucidate the bonding chemistry between the alkylamine and the GO after functionalization. The NMR results of GO–A18 investigation is presented in Figure 4. The peak at ~7 ppm is assigned to the CDCl_3_ solvent. The ^1^H NMR spectrum shows the presence of the alkylamine chain on the sample by several signals at 0.88 ppm (–CH_3_, small intensity) and 1.25 ppm (–CH_2_, very intense). It also showed a small peak around 3.14 ppm and 3.28 ppm which corresponds to the H peak of (–NH), which supports the reaction of the alkylamine with the GO [6,11,12]. Based on these results, it was confirmed that the γ-ray irradiation technique had successfully functionalized GO by the attachment of an alkylamine to the surface of the GO. The peak at ~1.56 ppm was due to moisture present in the sample. This moisture may have come from the NMR tube, the surrounding atmosphere, or the sample itself which may not have been completely dry. 

### 2.5. X-ray Photoelectron Spectroscopy

**Figure 5:**
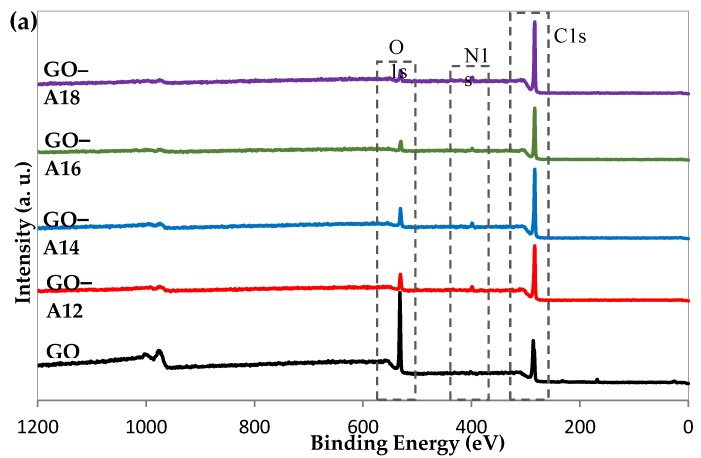

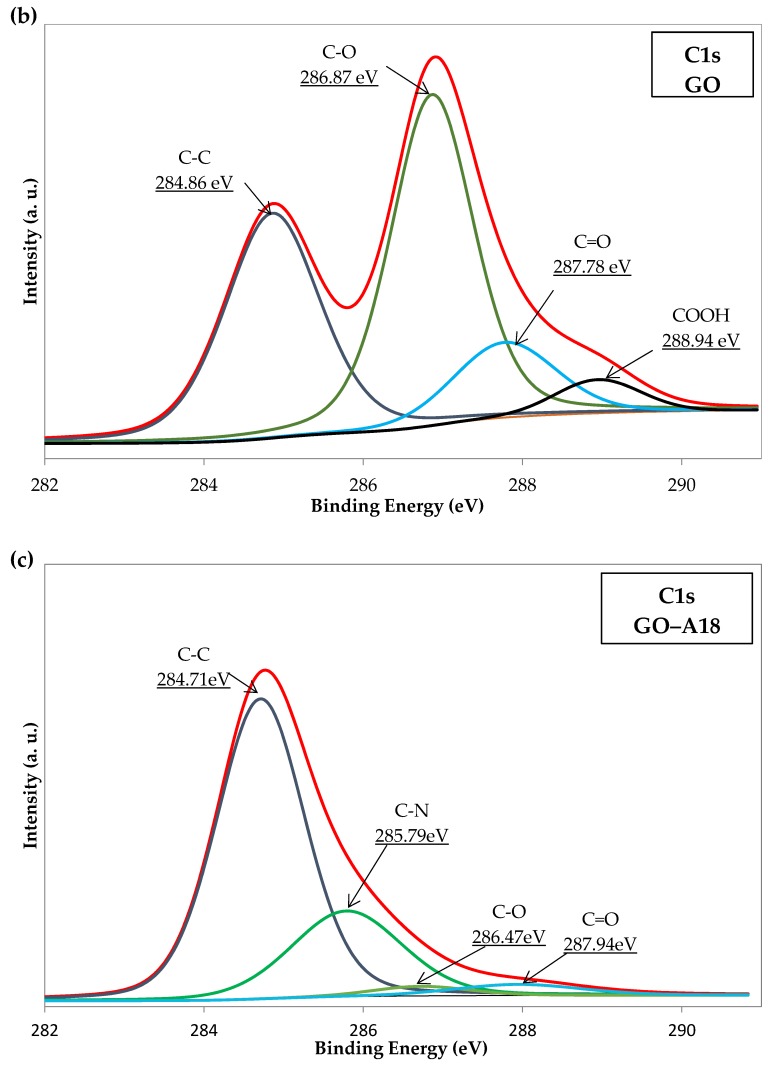

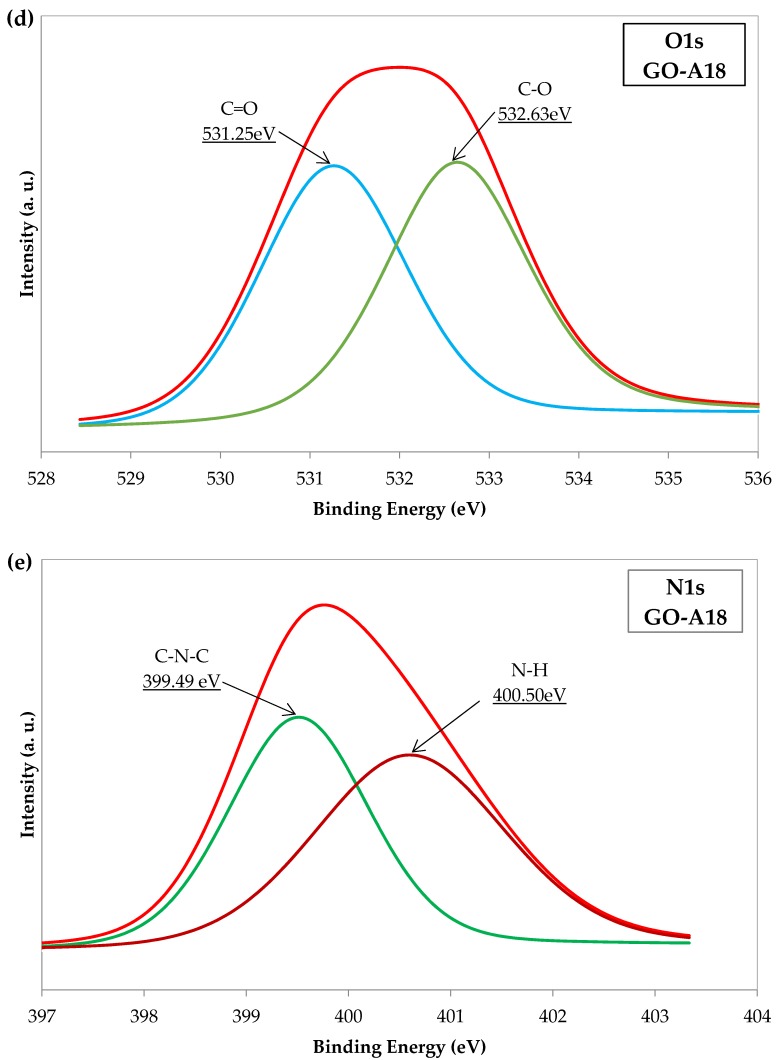
(**a**) XPS results of GO and functionalized-GO, and binding energy of (**b**) GO and (**c**–**e**) GO–A18.

Additionally, the successful reduction and functionalization is also proved by XPS analysis and the results are displayed in Figure 5. Survey scan results of the GO showed two strong peaks at 280 eV and 532 eV, which correspond to C1s and O1s peaks, respectively (Figure 5a). Functionalization of long alkylamines on the GO surface resulted in a significant increase in the intensity of the C1s peak (280 eV) with an extreme attenuation of the O1s peak (532 eV) which was also found by Zhang et al., [16]. The functionalization was also corroborated by the decrease in the O/C ratio from 2.34 (GO) to 0.30 (GO–A18), as shown in Table 2. Of the success of the functionalization is further revealed by the appearance of a new peak at 400 eV (N1s) (Figure 5a), which is further confirmed by the rise in the N/O ratio (Table 2). Deconvolution of the high-resolution spectra (C1s, O1s and N1s) was carried out and a few representative results are included in Figure 5b–e. The resolution of the XPS spectrometer does not allow the separate analysis of the C–C and C=C peaks, therefore it was treated as a single signal and compared with the peaks corresponding to the carbon atoms bound to other groups. Deconvolution of the C1s spectra of pristine GO showed four major peaks that correspond to C–C/C–H (284.79 eV), C–O (287.00 eV), C=O (288.62 eV) and COOH (290.51 eV). This indicates a considerable degree of oxidation of GO, which indicates the presence of new oxygen-containing functional groups in the GO structure [37]. In addition, the functionalization of GO with alkylamines such as octadecylamine (GO–A18) resulted in a significant rise in the C–C/C–H peak and a drastic decrease in the peaks corresponding to C–O, C=O and COOH, along with the appearance of a peak at 285.79 eV (C–N) [38]. The information provided by the analysis of the O1s spectra complements the information provided by the analysis of the C1s spectra. In comparison to the changes in the C1s peaks upon functionalization, there is sudden decrease of the C–O and C=O peaks, and hence only two peaks appeared in the deconvoluted O1s spectra. This fact is further revealed from the deconvoluted N1s spectra, which showed two peaks at 399.49 eV (C–N) and at 400.50 eV (N–H). 

### 2.6. Scanning Electron Microscopy (SEM)

**Figure 6:**
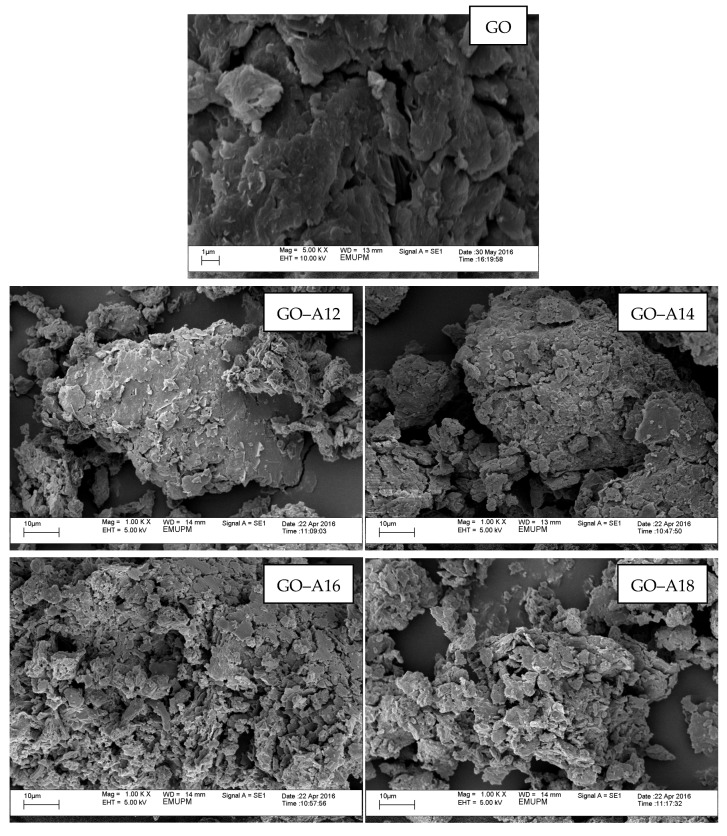
SEM microscopy of GO and functionalized GO.

The surface morphologies of the pristine GO and the functionalized GO were examined by using SEM and the micrographs are shown on Figure 6. The pristine GO showed the close stacking of the sheets with relatively smooth surfaces together with very sharp edges [35], compared to functionalized GO. However, the samples showed crumpled flakes with wrinkles and some folded regions in random orientations after functionalization with the alkylamine. For GO–A12 there were only a few aggregated domains that are less dense and the surface roughness is quite low. Increasing the chain length of the alkylamine (GO–A14, GO–A16 and GO–A18) resulted in the formation of many large and more obvious thick domains with high surface roughness indicated that these long-chain alkylamines were successfully attached on the GO surfaces. The difference in the surface roughness between GO and functionalized GO can be explained in terms of their chemical properties. GO is hydrophilic, which means it can easily disperse in water to form individual sheets with smooth surfaces due to the slow vacuum–filtration process. However, the attachment of long alkyl chains onto the GO changed it to a hydrophobic substance, leading to poor dispersion in a polar solvent. Functionalized GO also tended to form large aggregated domains due to a fast vacuum–filtration process. These special morphological features not only enhanced the surfaces roughness, but also substantially influenced the wettability properties of the solid surfaces and the dispersion behaviours of the samples [39,40,41].

## 3. Materials and Methods 

### 3.1. Materials 

Graphite powder (<20 µm) was obtained from Sigma Aldrich (St. Louis, MO, USA). Sulfuric acid (H_2_SO_4_, 98%) and hydrogen peroxide (H_2_O_2_, 30%) were obtained from R&M Chemicals (Selangor, Malaysia) and used as received. Potassium permanganate (KMnO_4_, 99.9%) and hydrochloric acid (HCl, 36%) were purchased from SYSTERM (Selangor, Malaysia). Dodecylamine, tetradecylamine, hexadecylamine and octadecylamine were purchased from Acros Organic (Geel, Belgium). All other chemicals were of analytical grade and were used as received without further purification. Distilled water was used for the washings.

### 3.2. Preparation of GO

Graphene oxide was synthesized from graphite using a simplified Hummers’ method. Five grams of graphite were first oxidized by reacting them with concentrated H_2_SO_4_ (500 mL) before adding KMnO4 (18 g). The reaction was allowed to proceed for 60 h to fully oxidize the graphite to graphite oxide. H_2_O_2_ solution (5% *v*/*v*) was added to destroy any excess KMnO_4_ and terminate the oxidation process. The GO formed was washed several times with a 1M aqueous HCl solution to remove any Mn^2+^ ions. Then, the mixture was washed repeatedly with distilled water to remove SO_4_^2−^ ions in the mixture. The washing process was carried out using a centrifugation technique. Water was added to the GO slurry for centrifugation at 9000 rpm, 20 min. The supernatant was decanted, leaving the GO slurry. The washing process was repeated several times. The final product was vacuum dried in a vacuum dryer at 80 °C and a pressure of 70 cm Hg.

### 3.3. Functionalization of GO

GO (0.6 g) was dissolved and exfoliated in 300 mL deionized water via ultrasonication. The resulting suspension was mixed with the solution of octadecylamine (0.9 g) in 90 mL ethanol. The mixture then underwent ultrasonication before being sent for irradiation treatment. The ^60^Co γ-ray source was used at room temperature with total doses of 25 kGy. After irradiation treatment, the resultant product (denoted as GO–A18) was separated by a filtration process. The collected solid was thoroughly washed with ethanol several times and dried at 70 °C in a vacuum oven. The dried GO–A18 was then ready for analysis. Similar synthesis steps were followed in preparing dodecylamine-(GO–A12), tetradecylamine-(GO–A14) and hexadecylamine-(GO–A16) functionalized GO.

### 3.4. Characterizations

Fourier Transform Infrared spectra were recorded using a Spectrum BX Perkin Elmer with the UATR technique (Waltham, MA, USA). The infrared spectra of the samples were recorded in the frequency range of 280–4000 cm^−1^ at 25 °C. A Perkin Elmer delta series TGA-7 (Waltham, MA, USA) was used for thermogravimetric analysis (TGA) of the samples. About 10–15 mg of each sample was used for the analysis. The samples were heated from 35 °C to 800 °C at a heating rate of 10 °C/min. The analysis was carried out in nitrogen at a flow rate of 20 mL/min. The weight-loss–temperature graph was plotted. The XRD with Cu Kα radiation (λ = 1.542 Å) operated at 30 kV and 30 mA and was used to determine the interlayer spacing of the GO sheets before and after functionalization. Data were collected within the range of scattering angles (2θ) of 2° to 30° at the rate of 2°/min. ^1^H NMR spectra were obtained from a JOEL JMTC spectrometer (500 MHz) (Peabody, MA, USA). The chemical shifts were recorded in ppm relative to a tetramethylsilane (TMS, 0.00 ppm) signal and deuterated chloroform (CD_3_Cl) was used as a solvent. X-ray photoelectron spectroscopy (XPS) was employed to investigate the surface chemical state of the GO and the functionalized GO. The analysis was performed on a ULVAC-PHI Quantera II (Ulvac-PHI, Inc., Kanagawa, Japan) instrument using a monochromatic Al-Kα (1486.6 eV) source at a power of 25.6 W. Scanning electron microscopy (SEM) images were performed at a magnification of 1000× by a JEOL JSM-6400F machine (Tokyo, Japan).

## 4. Conclusions

This work suggested that pristine graphite, when treated directly with appropriate chemicals, can readily generate exfoliated graphene oxide without the need for any chemical agents. This method does not generate toxic gas, in contrast to Hummers’ method. With further surface modifications, GO with varying properties is possible, thereby expanding the applications of GO. GO was successfully functionalized and slightly reduced by γ-ray irradiation in a mixed ethanol-water solution. The structural changes of the involved chemicals due to the radiation-induced functionalization were proved by means of XRD, FTIR, NMR, XPS and TGA. The elimination of the hydroxyl groups and the decarboxylation effect was demonstrated. Alkyl groups were attached onto the GO sheets due to the recombination of radicals. The SEM images show that functionalized GO acquired a high surface roughness. All the results indicate that the radiation-induced functionalization in ethanol/water is a good method to obtain functionalized GO, which is desirable for the preparation of hydrophobic materials.

## Figures and Tables

**Table 1:** Summary of GO synthesis methods.

Method	Year	Oxidants	Solvent	Reference
Brodie	1859	KClO_3_	HNO_3_	[19]
Staudenmaier	1898	KClO_3_	HNO_3_ (fuming), H_2_SO_4_	[20]
Hofmann	1937	KClO_3_	HNO_3_, H_2_SO_4_	[21]
Hummers	1958	NaNO_3_, KMnO_4_	H_2_SO_4_	[22]
Modified Hummers	1999	K_2_S_2_O_8_, P_2_O_5_, KMnO_4_	H_2_SO_4_	[23]
	2010	KMnO_4_	H_2_SO_4_, H_3_PO_4_	[24]

**Table 2:** Survey scan results for functionalized GO samples.

Sample	O/C Ratio	N/O Ratio
GO	2.34	0.08
GO–A12	0.51	0.91
GO–A14	0.48	0.74
GO–A16	0.36	0.74
GO–A18	0.30	1.55

## References

[1] Hernandez Y., Nicolosi V., Lotya M., Blighe F.M., Sun Z., De S., McGovern I.T., Holland B., Byrne M., Gun’Ko Y.K. (2008). High-yield production of graphene by liquid-phase exfoliation of graphite. Nat. Nanotechnol..

[2] Choucair M., Thordarson P., Stride J.A. (2009). Gram-scale production of graphene based on solvothermal synthesis and sonication. Nat. Nanotechnol..

[3] Lerf A., He H., Forster M., Klinowski J. (1998). Structure of Graphite Oxide Revisited. J. Phys. Chem. B.

[4] Park S., Ruoff R.S. (2009). Chemical methods for the production of graphenes. Nat. Nanotechnol..

[5] Stankovich S., Piner R.D., Nguyen S.T., Ruoff R.S. (2006). Synthesis and exfoliation of isocyanate-treated graphene oxide nanoplatelets. Carbon.

[6] Li W., Tang X.-Z., Zhang H.-B., Jiang Z.-G., Yu Z.-Z., Du X.-S., Mai Y.-W. (2011). Simultaneous surface functionalization and reduction of graphene oxide with octadecylamine for electrically conductive polystyrene composites. Carbon.

[7] Cao Y., Feng J., Wu P. (2010). Alkyl-functionalized graphene nanosheets with improved lipophilicity. Carbon.

[8] Compton O.C., Dikin D.A., Putz K.W., Brinson L.C., Nguyen S.T. (2010). Electrically Conductive “Alkylated” Graphene Paper via Chemical Reduction of Amine-Functionalized Graphene Oxide Paper. Adv. Mater..

[9] Mei Q., Zhang K., Guan G., Liu B., Wang S., Zhang Z. (2010). Highly efficient photoluminescent graphene oxide with tunable surface properties. Chem. Commun..

[10] Guan W., Li Z., Zhang H., Hong H., Rebeyev N., Ye Y., Ma Y. (2013). Amine modified graphene as reversed-dispersive solid phase extraction materials combined with liquid chromatography–tandem mass spectrometry for pesticide multi-residue analysis in oil crops. J. Chromatogr. A.

[11] Zhang S.P., Song H.O. (2012). Supramolecular graphene oxide-alkylamine hybrid materials: Variation of dispersibility and improvement of thermal stability. New J. Chem..

[12] Shanmugharaj A.M., Yoon J.H., Yang W.J., Ryu S.H. (2013). Synthesis, characterization, and surface wettability properties of amine functionalized graphene oxide films with varying amine chain lengths. J. Colloid Interface Sci..

[13] Gupta B., Kumar N., Panda K., Melvin A.A., Joshi S., Dash S., Tyagi A.K. (2016). Effective Noncovalent Functionalization of Poly(ethylene glycol) to Reduced Graphene Oxide Nanosheets through γ-Radiolysis for Enhanced Lubrication. J. Phys. Chem. C.

[14] Li J., Zhang B., Li L., Ma H., Yu M., Li J. (2014). γ-ray irradiation effects on graphene oxide in an ethylenediamine aqueous solution. Radiat. Phys. Chem..

[15] Dumée L.F., Feng C., He L., Yi Z., She F., Peng Z., Gao W., Banos C., Davies J.B., Huynh C. (2014). Single step preparation of meso-porous and reduced graphene oxide by gamma-ray irradiation in gaseous phase. Carbon.

[16] Zhang B., Li L., Wang Z., Xie S., Zhang Y., Shen Y., Yu M., Deng B., Huang Q., Fan C. (2012). Radiation induced reduction: An effective and clean route to synthesize functionalized graphene. J. Mater. Chem..

[17] Safibonab B., Reyhani A., Nozad Golikand A., Mortazavi S.Z., Mirershadi S., Ghoranneviss M. (2011). Improving the surface properties of multi-walled carbon nanotubes after irradiation with gamma rays. Appl. Surf. Sci..

[18] Miao M., Hawkins S.C., Cai J.Y., Gengenbach T.R., Knott R., Huynh C.P. (2011). Effect of gamma-irradiation on the mechanical properties of carbon nanotube yarns. Carbon.

[19] Brodie B.C. (1859). On the Atomic Weight of Graphite. Philos. Trans. R. Soc. Lond..

[20] Staudenmaier L. (1898). Verfahren zur Darstellung der Graphitsäure. Ber. Dtsch. Chem. Ges..

[21] Hofmann U., König E. (1937). Untersuchungen über Graphitoxyd. Z. Anorg. Allg. Chem..

[22] Hummers W.S., Offeman R.E. (1958). Preparation of Graphitic Oxide. J. Am. Chem. Soc..

[23] Kovtyukhova N.I., Ollivier P.J., Martin B.R., Mallouk T.E., Chizhik S.A., Buzaneva E.V., Gorchinskiy A.D. (1999). Layer-by-Layer Assembly of Ultrathin Composite Films from Micron-Sized Graphite Oxide Sheets and Polycations. Chem. Mater..

[24] Marcano D.C., Kosynkin D.V., Berlin J.M., Sinitskii A., Sun Z., Slesarev A., Alemany L.B., Lu W., Tour J.M. (2010). Improved Synthesis of Graphene Oxide. ACS Nano.

[25] Chen J., Yao B., Li C., Shi G. (2013). An improved Hummers method for eco-friendly synthesis of graphene oxide. Carbon.

[26] Chen J., Li Y., Huang L., Li C., Shi G. (2015). High-yield preparation of graphene oxide from small graphite flakes via an improved Hummers method with a simple purification process. Carbon.

[27] Wang C., Liu Z., Wang S., Zhang Y. (2016). Preparation and properties of octadecylamine modified graphene oxide/styrene-butadiene rubber composites through an improved melt compounding method. J. Appl. Polym. Sci..

[28] Gao W., Gao W. (2015). The Chemistry of Graphene Oxide. Graphene Oxide: Reduction Recipes, Spectroscopy, and Applications.

[29] Li Z., Zhang W., Luo Y., Yang J., Hou J.G. (2009). How Graphene Is Cut upon Oxidation?. J. Am. Chem. Soc..

[30] Chen Y., Tao J., Ezzeddine A., Mahfouz R., Al-Shahrani A., Alabedi G., Khashab N. (2015). Superior Performance Nanocomposites from Uniformly Dispersed Octadecylamine Functionalized Multi-Walled Carbon Nanotubes. C J. Carbon Res..

[31] Yan J.-L., Chen G.-J., Cao J., Yang W., Xie B.-H., Yang M.-B. (2012). Functionalized graphene oxide with ethylenediamine and 1,6-hexanediamine. New Carbon Mater..

[32] Guerrero-Contreras J., Caballero-Briones F. (2015). Graphene oxide powders with different oxidation degree, prepared by synthesis variations of the Hummers method. Mater. Chem. Phys..

[33] Dumée L.F., Feng C., He L., Allioux F.-M., Yi Z., Gao W., Banos C., Davies J.B., Kong L. (2014). Tuning the grade of graphene: Gamma ray irradiation of free-standing graphene oxide films in gaseous phase. Appl. Surf. Sci..

[34] Ansón-Casaos A., Puértolas J.A., Pascual F.J., Hernández-Ferrer J., Castell P., Benito A.M., Maser W.K., Martínez M.T. (2014). The effect of gamma-irradiation on few-layered graphene materials. Appl. Surf. Sci..

[35] Zhu C., Guo S., Fang Y., Dong S. (2010). Reducing Sugar: New Functional Molecules for the Green Synthesis of Graphene Nanosheets. ACS Nano.

[36] Choudhary S., Mungse H.P., Khatri O.P. (2012). Dispersion of alkylated graphene in organic solvents and its potential for lubrication applications. J. Mater. Chem..

[37] Sobon G., Sotor J., Jagiello J., Kozinski R., Zdrojek M., Holdynski M., Paletko P., Boguslawski J., Lipinska L., Abramski K.M. (2012). Graphene Oxide vs. Reduced Graphene Oxide as saturable absorbers for Er-doped passively mode-locked fiber laser. Opt. Express.

[38] Ryu S.H., Shanmugharaj A.M. (2014). Influence of long-chain alkylamine-modified graphene oxide on the crystallization, mechanical and electrical properties of isotactic polypropylene nanocomposites. Chem. Eng. J..

[39] Ma W.-S., Li J., Deng B.-J., Zhao X.-S. (2013). Preparation and characterization of long-chain alkyl silane-functionalized graphene film. J. Mater. Sci..

[40] Bao H., Pan Y., Ping Y., Sahoo N.G., Wu T., Li L., Li J., Gan L.H. (2011). Chitosan-Functionalized Graphene Oxide as a Nanocarrier for Drug and Gene Delivery. Small.

[41] Carreira L., Mendes L., Ribeiro M., Sebastião P. (2013). Organically-Expanded Graphite/Octadecylamine: Structural, Thermal and Relaxation Evaluation. Mater. Sci. Appl..

